# Delineation of gastrointestinal tumors biopsies using a fluorescence lifetime imaging optical fiber probe

**DOI:** 10.1002/jbio.202400122

**Published:** 2024-07-16

**Authors:** D. Suraci, E. Baria, L. Tirloni, J. L. Lagarto, S. Buccianti, C. Agostini, S. Pillozzi, L. Antonuzzo, A. Taddei, R. Cicchi

**Affiliations:** ^1^ European Laboratory for Non‐linear Spectroscopy (LENS) Sesto Fiorentino Italy; ^2^ National Institute of Optics National Research Council (CNR‐INO) Florence Italy; ^3^ Department of Physics University of Florence Sesto Fiorentino Italy; ^4^ Hepatobiliopancreatic Surgery Careggi University Hospital Florence Italy; ^5^ Biophotonics Platform Champalimaud Foundation Lisbon Portugal; ^6^ Department of Experimental and Clinical Biomedical Sciences 'Mario Serio' University of Florence Florence Italy; ^7^ Clinical Oncology Unit Careggi University Hospital Florence Italy; ^8^ Department of Experimental Clinical Medicine University of Florence Florence Italy

**Keywords:** augmented reality, autofluorescence, fiber optics, FLIM, fluorescence lifetime imaging, fluorescence spectroscopy, real‐time imaging, surgical guidance, TCSPC, tissue interrogation

## Abstract

Autofluorescence spectroscopy has emerged in recent years as a powerful tool to report label‐free contrast between normal and diseased tissues, both in vivo and ex‐vivo. We report the application of an instrument employing an optical fiber probe and capable of performing real‐time autofluorescence lifetime imaging at a macroscopic scale, under bright background conditions. We validate and demonstrate the practicality of this technology to discriminate healthy against neoplastic tissue in freshly excised tumor biopsies. The capability of delineating tumor margins through processing the fluorescence decays in the phasors domain was demonstrated on four different types of cancer, highlighting the broad range of potential clinical applications for the proposed approach. The presented results suggest that our autofluorescence lifetime imaging probe, together with phasor analysis, can offer a real‐time tool to observe lifetime contrast on tissues and, thus, is a suitable candidate for improving in situ tissue diagnostics during surgery.
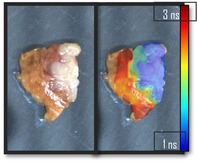

## Graphical abstract

The efficient discrimination of a hepatic cancer lesion and its surgical margin using autofluorescence lifetime imaging is presented. White light image of the specimen on the left and the augmented image with fluorescence lifetime data on the right. Our optical fiber‐based setup opens new possibilities for using this technology in surgeries across different cancer types.

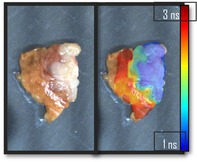

## 1 INTRODUCTION

Cancer remains a major global health challenge, with several new cases and deaths reported every year. In 2023, 1 958 310 new cancer cases are projected to occur only in the United States [1, 2] and 609 820 of these will lead to death. Complete, margin‐negative resection (where some healthy tissue is removed to ensure the whole tumor is cut out) is essential to increase chances of long‐term survival in various types of tumors (pancreatic cancer, primary liver cancer, and colorectal cancer) [3, 4, 5, 6, 7, 8, 9, 10]. However, such extensive resections, especially when they involve nearby blood vessels and/or organs, should only be undertaken if the pre‐surgical imaging indicates the possibility to achieve a complete, margin‐negative resection. On the other hand, very often surgical margins are assessed to achieve a negative resection during surgery [11], upon processing and histopathological examination of freshly excised tissue specimens, leading to a prolonged operation time and possible associated complications [12]. In this scenario, it is of paramount importance a rapid, accurate and safe method for the successful intrasurgical diagnosis and management of these diseases.

The landscape of noninvasive diagnostic techniques for early detection of cancerous diseases has witnessed remarkable advancements with the emergence of optical imaging technologies [13, 14, 15, 16]. Recent years have observed significant steps in the development of optical label‐free diagnostic alternatives to standard medical imaging modalities. Among these techniques, Fluorescence Lifetime Imaging Microscopy (FLIM) and Time‐Resolved Fluorescence Spectroscopy (TRFS) have shown promising potential in discriminating diseased against healthy tissue with the potential for translation from the research labs to bedside. These cutting‐edge technologies, operating in a label‐free modality, offer detailed profiles of tissue autofluorescence, thereby enabling more informed and rapid tissue characterization. In fact, autofluorescence lifetime imaging (FLIm), leverages the optical properties of endogenous fluorophores, such as collagens, nicotinamide adenine dinucleotide (phosphate) (NAD(P)H), flavin adenine dinucleotide (FAD) and flavin mononucleotide (FMN), to provide label‐free contrast and report on the structural and functional properties of cells and tissues at various stages of the disease [13, 17, 18, 19]. In particular the primary regulators of cellular redox state are the redox couples nicotinamide adenine dinucleotide (NAD+/NADH) and nicotinamide adenine dinucleotide phosphate (NADP+/NADPH). Despite their crucial roles, they participate in separate metabolic pathways. NAD drives ATP production both in the cytosol via glycolysis and in the mitochondria through oxidative phosphorylation. Conversely, NADP, in its phosphorylated form, oversees pathways involved in lipid, amino acid, and nucleotide biosynthesis, as well as the defense against reactive oxygen species through glutathione (GSH) [20]. In this scenario, the autofluorescence lifetime measurements offer insights into metabolic mechanisms associated with abnormal cellular function and proliferation by targeting NAD(P)H. It has been already demonstrated that fluorescence spectroscopy of endogenous molecules, and more specifically autofluorescence imaging, can be used in oncology diagnostic for discriminating cancerous from healthy cells [21, 22]. Time‐correlated single‐photon counting (TCSPC) is recognized as the “gold‐standard” method for fluorescence lifetime measurements owing to its temporal resolution, sensitivity, photon efficiency, dynamic range, and capability to resolve complex decay functions [23, 24, 25]. A major limitation of TCSPC refers to its inability to distinguish fluorescence photons from background photons, which makes TCSPC measurements impractical under bright background light conditions. This limitation can be circumvented by means of an asynchronous detection of the fluorescence signal with respect to an external white light source, as described in our previous implementation [26].

Here we report the test of our autofluorescence lifetime imaging probe device, already employed for real‐time mapping of degraded articular cartilage from pig [26, 27], on four different clinical cases that required surgery as fundamental therapeutic approach, to demonstrate that autofluorescence lifetime is a valid tool for rapid characterization and delineation of healthy surgical margins with respect to the cancer mass. We examined two liver biopsies, one derived from a hepatocellular carcinoma (HCC) resection and the other from an intrahepatic cholangiocarcinoma (ICC), a sample of gastrointestinal stromal tumor (GIST) and a sample of pancreatic ductal adenocarcinoma (PDCA). The autofluorescence lifetime maps obtained from liver and gastrointestinal specimens can assess the differences over the surgical margin, affirming the potential of our fiber‐based platform for real‐time margin assessment and delineation. Slightly smaller changes were observed in PDCA when comparing the autofluorescence lifetime data of healthy tissue against tumor tissue, but still enough to discriminate tumor borders, based on the autofluorescence lifetime map. The phasors approach for data analysis opens the possibility to the surgical use of the method, thanks to the real‐time processing and visual feedback offered to the clinician. This result, together with the successful test of our fiber‐based optical setup for margin assessment across four clinical cases, opens the door to the potential application of the system to various clinical diagnostics applications, including tumor margin delineation, treatment response monitoring and post‐surgery residual tumor detection.

## 2 EXPERIMENTAL

### 2.1 Sample collection and preparation

Surgical specimens and biopsies from various organs and tumors, with sizes ranging from 1 to 3 cm, were provided by the surgeon team of Hepatobiliopancreatic Surgery (Careggi University Hospital). These samples, including two cases of liver cancer (one HCC and an ICC), one instance of a GIST, and one case of PDAC, were obtained from patients who underwent tumor resection procedures during the year 2023. The study protocol was approved by the Careggi University Hospital Ethics Committee (approval number: 21913_bio) and written informed consent was obtained from all study participants prior to participation. All procedures were performed in accordance with the recommendations of the Declaration of Helsinki. Four patients have been involved in this study (one per pathology), with an average age of 70 years. A dedicated specialist evaluated the pathological profile of these lesions before acquiring autofluorescence data. Each tissue sample was meticulously recognized and annotated in three distinct regions: the tumor lesion, the marginal tissue surrounding the lesion, and the healthy tissue located at remotely from the lesion. For the PDAC sample, the collection of a corresponding healthy specimen was prevented by clinical constraints. The ex vivo tissue samples underwent an initial cleansing process in Dulbecco's phosphate‐buffered saline (DPBS) at a temperature of 4°C to eliminate any residual blood or tissue clumps. Subsequently, the tissues were positioned on sterile petri dishes (60 × 15 mm) for the autofluorescence measurements. Throughout the entire experimental process, the samples were maintained in PBS at a temperature of 4°C, except during spectroscopic measurements, performed at room temperature. All the acquisitions were performed within 30 min from the excision. We verified that the photon counts remained constant for about 40 min from excision in order to present homogenous data in terms of photon counts.

### 2.2 Experimental setup

**FIGURE 1:**
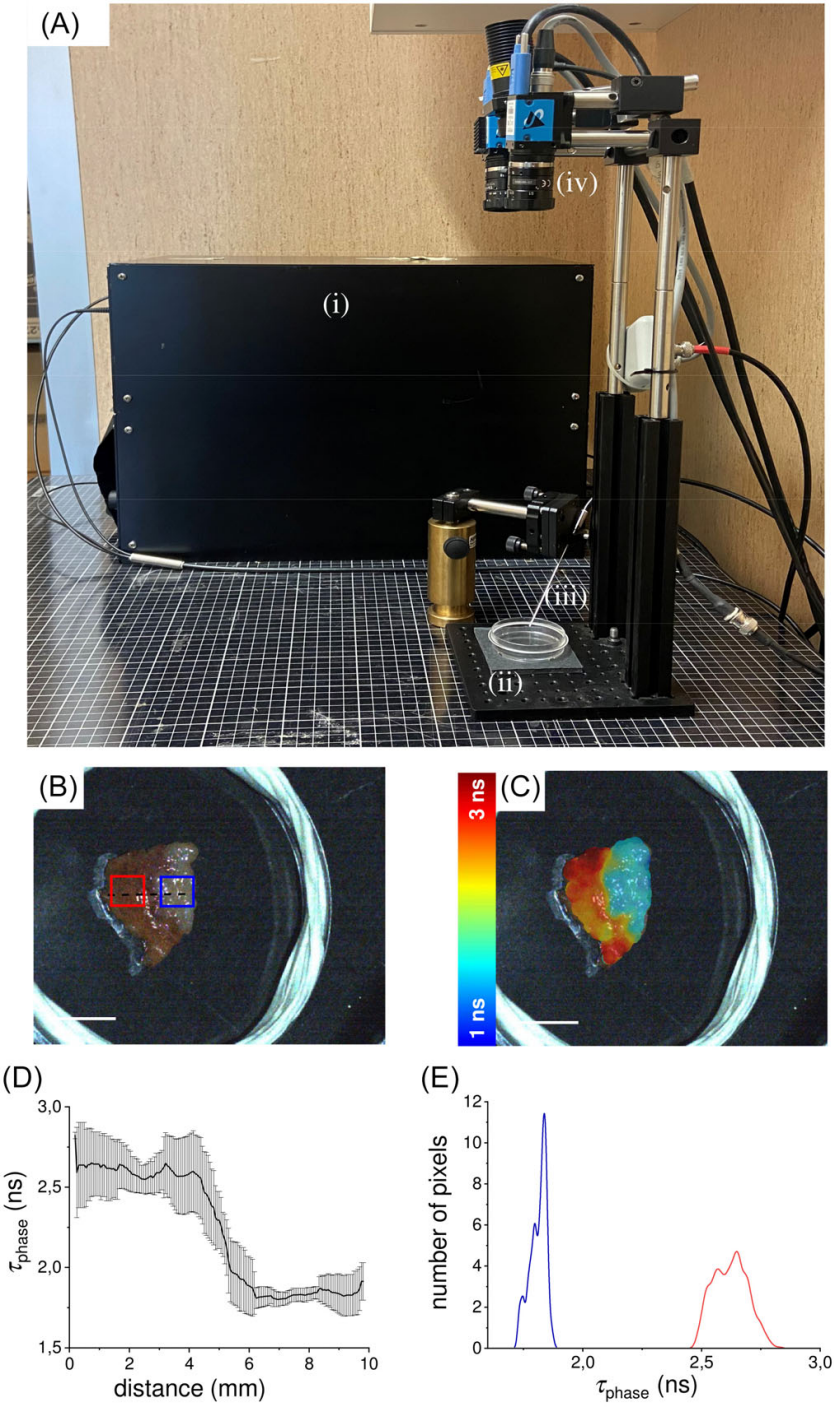
(A) Optical setup. Photo of the experimental setup used for autofluorescence lifetime imaging measurements, showing: (i) the black metallic box containing the laser source, optical and optomechanical components, filters, drivers, electronics as well as the hybrid detector; (ii) the sample holder; (iii) the optical fiber probe; (iv) the bright field system consisting of a white LED and a color camera. Examples of a measurement on a liver metastasis showing: (B) the white light image where the healthy tissue is brown‐red (red ROI) and the cancer lesion is light brown (blue ROI); the corresponding augmented‐reality image (C), made by merging the white light image with fluorescence lifetime data represented in a color‐coded scale. Scale bar = 10 mm; (D) the autofluorescence lifetime profile of the median region between the margin and the cancer lesion (dashed black line in b); (E) the autofluorescence lifetime distribution within the two ROIs in (b): the cancer (blue line), the perilesional tissue (red line). Hybrid detector (HPM‐100‐40, Becker & Hickl GmbH), connected to a TCSPC acquisition card (SPC‐730, Becker & Hickl GmbH). A white light‐emitting diode (MNWHL4, Thorlabs), square‐wave modulated at a frequency of 50 Hz with an ‘on’ time of 2 ms, was employed to provide periodic illumination of the field of view (FOV). At this frequency, the stroboscopic effect produced by the LED's on–off switching is not perceived by the human eye, resulting in a continuous illumination of the FOV for the operator. A USB color camera with a resolution of 640 × 480 pixels (DFK33UP1300, The Imaging Source, Bremen, Germany) was used to record the measurements and for correlating each autofluorescence decay point with a specific position on the specimen. Then, lifetime maps are generated using phasors analysis of the autofluorescence decays and superimposed as augmented reality on the white light image, providing real‐time visual feedback to the operator during data acquisition.

The optical setup employed in this study, which has been exhaustively described elsewhere [26, 28], featured a picosecond pulsed laser diode emitting light at 375 nm (BDL‐SMN‐375, Becker & Hickl GmbH, Berlin, Germany) with a repetition rate of 20 MHz. The excitation light, together with a superimposed 660 nm aiming beam provided by a red light‐emitting diode (M660FP1, Thorlabs, Newton, NJ, USA), was delivered to the samples through a spliced optical fiber with a core diameter of 400 μm (NA 0.22), that is part of a custom‐made trifurcated optical fiber bundle (FiberTech Optica, Montreal, Canada). This fiber bundle consists of one central optical fiber for excitation and nine collection 200 μm optical fibers that are arranged in a concentric ring around the delivery fiber at the distal end and grouped within an SMA connector at the proximal end. The autofluorescence signal emerging from the fiber bundle was collimated, filtered by alternatively using two different emission filters (FF01‐470/28–25 and FF01‐534/20–25, Semrock, Rochester, NY, USA), and then directed to a fast‐response high‐sensitive (Figure 1).

### 2.3 Fluorescence data acquisitions

Autofluorescence lifetime acquisitions were performed with the tip of the fiber bundle handheld, allowing it to be maneuvered freely over the sample surface, while the aiming beam provided a visual reference for guiding the operator during data acquisition. The fiber probe was consistently maintained at approximately 3 mm from the sample and the excitation laser was kept at an average power below 20 μW during data acquisition. For these measurements, the surface of the specimens was mapped in approximately 45 s. The fiber was moved across the samples, scanning each point several times to produce smoother fluorescence lifetime maps, upon pixel averaging. This effectively smooths out the measured fluorescence lifetimes across the sampled area. System response de‐convolution and fluorescence decay analysis on image pixels were conducted using a phasor model in LabVIEW (LabVIEW 2015, National Instruments, Austin, TX, USA), which also allowed the presentation of data as augmented map in real‐time. The average number of photons collected in each acquisition was sufficiently high to obtain an accurate lifetime contrast in regions with different cellular metabolic conditions.

### 2.4 Fluorescence lifetime data analysis

Autofluorescence lifetime data were analyzed using phasors. Differently from fit‐based methods, the phasors analysis allows identifying pixel‐based lifetime characteristics without prior knowledge of the exact contribution of different components [29, 30]. The application of the phasor approach is largely described in literature [26, 28, 31] and its fit‐free nature is suitable for an implementation that requires a real‐time output, as it is based on an extremely fast processing algorithm, namely the FFT.

Briefly, for single‐exponential emitters, the phasor would align with the universal circle, centered at (*g* = 0.5, *s* = 0) with a radius of 0.5. Mixtures of two fluorophores would reside within this circle, representing a blend of their individual characteristics. The phase lifetime (*τ*‐phase) was calculated from *g* and *s* coordinates using Equation (1):

(1) $$\tau_{\text{phase}}=\frac{1}{\omega }\frac{s}{g}.$$

The decay curve is a sum of multiple components as each pixel represents an overlay of emissions from various fluorophores. The Instrument Response Function (IRF) was determined by measuring back‐reflected excitation light, which served as a reference for both calculation methods. Image processing and videos were realized using a custom‐written MATLAB application. For statistical analysis, a paired student's *t*‐test was applied to compare data from normal and from diseased tissue. All statistical analyses, graphs, and histograms were conducted and realized using Microcal Origin Pro 8.0 (OriginLab Corporation, Northampton, MA, US). Results were considered statistically significant with a *p*‐value <.01.

## 3 RESULTS AND DISCUSSION

### 3.1 Liver carcinoma

**FIGURE 2:**
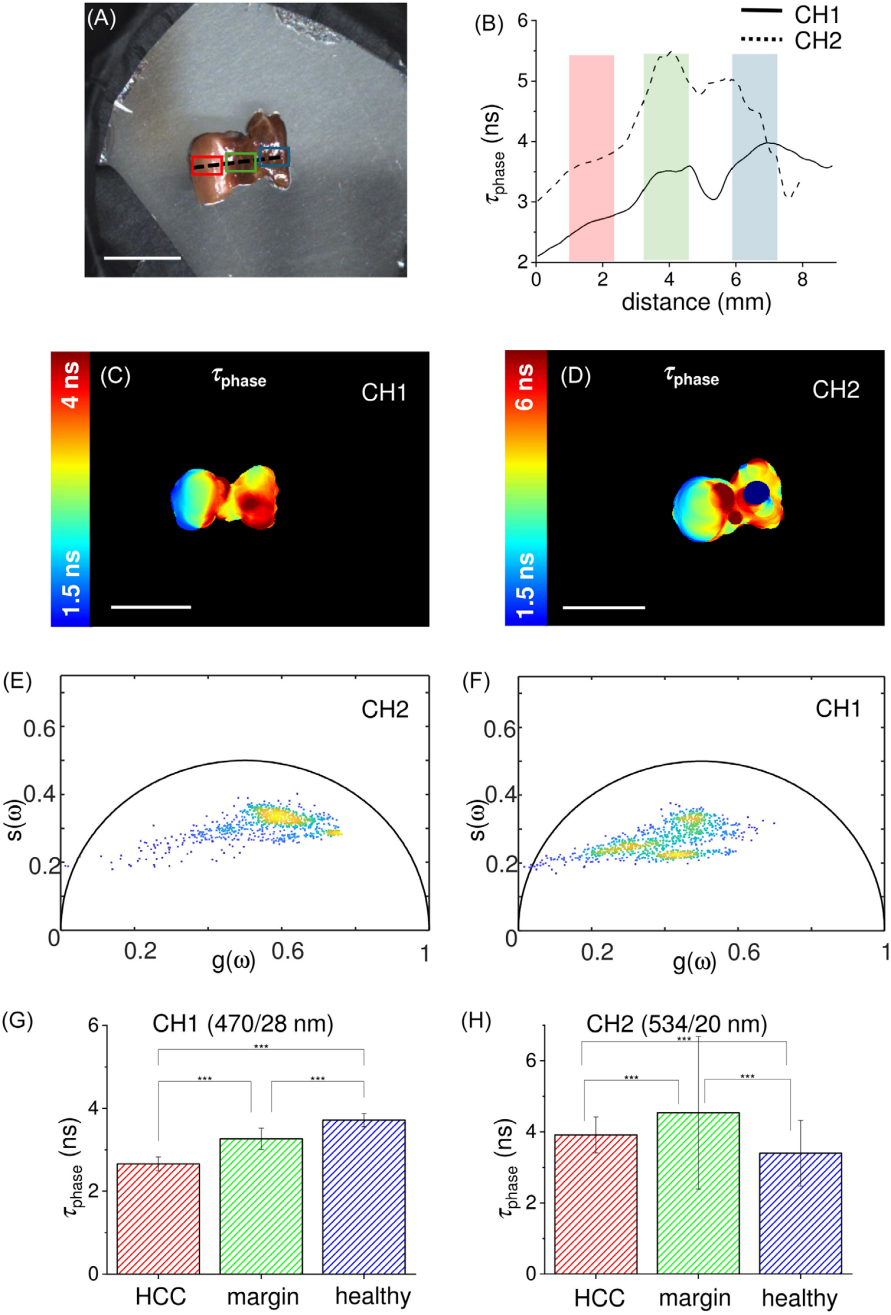
(a) White light image of the HCC sample with superimposed drawings of healthy tissue ROI (cyan), surgical margin ROI (green), tumor ROI (red), and ROIs crossing profile (black dashed line). (b) Profile of the autofluorescence lifetime values of *τ*_phase_ measured along the crossing profile in (a) for the channel 470/28 nm (CH1, black line) and 534/20 nm (CH2, black dashed line). Autofluorescence lifetime maps for the channel 470/28 nm (CH1, c) and 534/20 nm (CH2, d), with the corresponding phasor plots for the channel 470/28 nm (CH1, e) and 534/20 nm (CH2, f). Scale bars = 10 mm. (g, h) Histograms of the autofluorescence lifetime values of *τ*_phase_ acquired in the two spectral channels and averaged over ROI of tumor region (dark grey), surgical margin (light grey) and healthy tissue (grey dashed lines). Values are reported as mean ± standard deviation, and the student *t*‐test show the statistically significant differences between those ROIs (*p*‐values < .001).

The first reported ex vivo specimen was a tissue sample of HCC collected and measured within 30 min from excision. Three regions of interests (ROIs) were annotated, the tumor lesion, the healthy margin and healthy liver. Those regions are clearly recognizable looking at the white light image in Figure 2A. The HCC appears with a light brown shade on the left, whereas the surgical margin (on the centre) maintains the characteristic dark reddish‐brown associated with the liver tissue (on right side of the sample). The corresponding autofluorescence lifetime augmented maps shown in Figure 2C,D highlights the noticeable border. Autofluorescence lifetime measurements are presented for two distinct spectral bands: channel 1 (CH1), 470/28 nm (Figure 2C); channel 2 (CH2), 534/20 nm (Figure 2D). The surgical margin exhibits longer autofluorescence lifetime with respect to the HCC, clearly distinguishable from the autofluorescence lifetime maps. The corresponding phasor plots, respectively, reported in Figure 2E,F for CH1 and CH2, show three distinct clouds, evidencing the discrimination capability among the three tissue types, based on their autofluorescence lifetime. In Figure 2C, the three tissue types are depicted in blue/light‐blue, yellow, and dark red, demonstrating a lifetime increase when moving from the left to the right side of the image. A similar increase, when moving from the HCC to the margin, is shown in the lifetime map of CH2, represented in Figure 2D. Differently from CH1, where the autofluorescence lifetime further increases when moving from the margin to the healthy tissue, a slight decrease is observed in CH2. This datum is further confirmed by looking at the autofluorescence lifetime profiles reported in Figure 2B, where the behaviors of the autofluorescence lifetime along the dashed line in Figure 2A, are plotted for both CH1 (plain line) and CH2 (dashed line). In particular, we observed a general difference in lifetime values between the two channels, while they maintained a consistent trend when comparing surgical margin and tumor lesion, with a longer lifetime measured in the margin ROI with respect to the tumor ROI.

On the other hand, an opposite trend between the two channels is found when comparing surgical margin and healthy tissue, with healthy tissue showing a longer lifetime in CH1 and a shorter lifetime in CH2 when compared to the corresponding values measured in the margin. The same trend is observed when extending the data analysis from the profile to the whole ROIs, as reported in the histograms in Figure 2G for CH1 and 2 h for CH2. In detail, the average weighted lifetime (*τ*_phase_) of the signal in CH1 with 375 nm excitation (mostly NAD(P)H as fluorophore contribution) was 2.7 ± 0.2 ns, whereas the *τ*_phase_ corresponding to CH2 was 3.9 ± 0.5 ns. The margin region presented a *τ*_phase_ of 3.2 ± 0.2 ns in CH1, while in CH2 the value increases up to 4.5 ± 2 ns. Healthy tissue shows a *τ*_phase_ of 3.7 ± 0.2 ns in CH1, face to a value of 3.4 ± 0.9 ns in CH2 that is lower than CH1, differently from what has been found in the other ROIs. Autofluorescence lifetime values, calculated as average of the examined ROIs, are reported in detail in Table 1, divided by lesion and ROI type.

**TABLE 1:** Autofluorescence lifetime values expressed as τ_phase_ (±standard deviation), divided by lesion type and calculation method, and averaged within different ROIs.

Lesion	ROIs	*τ*_phase_ CH1 (ns)	SD	*τ*_phase_ CH2 (ns)	SD	Histopathology
HCC	Healthy	3.7	0.1	3.4	1.0	Trabecular cell architecture ES grade I T1b CD34 +
	Margin	3.3	0.2	4.6	2.0	
	Tumor	2.6	0.2	3.9	0.5	
ICC	Healthy	2.2	0.1	2.3	0.3	Combined Hepatocellular‐Cholangiocarcinoma HCC‐CCA tumor CK7 +, CK20 +, HAS +
	Margin	2.1	0.2	2.1	0.2	
	Tumor	4.2	0.2	4.6	0.3	
GIST	Healthy	2.1	0.1	1.8	0.1	Gastrointestinal tumor mostly spindle cells
	Margin	2.1	0.1	1.7	0.1	
	Tumor	2.4	0.1	2.3	0.1	
PDCA	Margin	4.4	0.5	4.4	0.6	Papillary adenocarcinoma, pancreato‐biliary (PB) moderated grade T3aN0
	Tumor	3.5	0.1	4.9	0.2	

**FIGURE 3:**
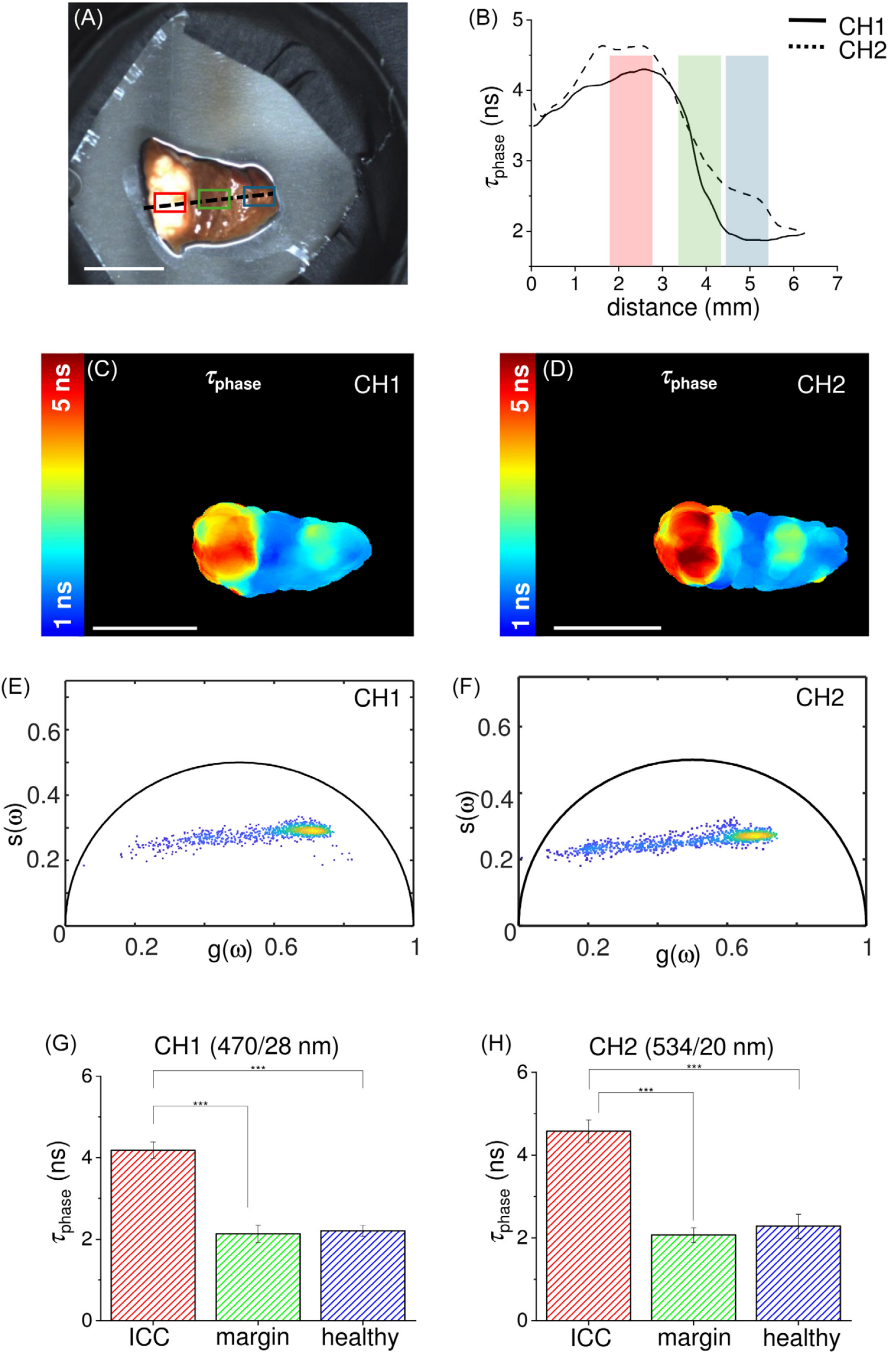
(a) White light image of the ICC sample with superimposed drawings of healthy tissue ROI (cyan), surgical margin ROI (green), tumor ROI (red), and ROIs crossing profile (black dashed line). (b) Profile of the autofluorescence lifetime values of *τ*_phase_ measured along the crossing profile in (a) for the channel 470/28 nm (CH1, black line) and 534/20 nm (CH2, black dashed line). Autofluorescence lifetime maps for the channel 470/28 nm (CH1, c) and 534/20 nm (CH2, d), with the corresponding phasor plots for the channel 470/28 nm (CH1, e) and 534/20 nm (CH2, f). Scale bars = 10 mm. (g, h) Histograms of the autofluorescence lifetime values of *τ*_phase_ acquired in the two spectral channels and averaged over ROI of tumor region (dark grey), surgical margin (light grey) and healthy tissue distant from the lesion (grey dashed lines). Values are reported as mean ± standard deviation, and the student *t*‐test show the statistically significant differences between those ROIs (*p*‐values <.001).

In Figure 3, a different sample of liver cancer is presented, specifically a cholangiocarcinoma (ICC). Like the HCC, the ICC is identifiable by looking at the white light image [32, 33] (Figure 3A), and three ROIs with three different colours (cyan for healthy, green for margin, red for tumor) were meticulously annotated.

The light‐brown region on the left side of the sample is the ICC location, while in the center and on the right side we have the surgical margin and the healthy tissue distant from the tumor, with their typical dark‐red colour. In this example, differently from the previous on HCC, the surgical margin exhibits shorter autofluorescence lifetime with respect to the tumor, clearly distinguishable from the autofluorescence lifetime maps (Figure 3C,D for CH1 and CH2, respectively). The corresponding phasor plots, Figure 3E,F for CH1 and CH2, show a very similar behavior for the two detection channels, with phasor points that are spread along a line within the universal circle. Although three distinct clouds are not immediately distinguishable, the distribution presents three regions with high density of phasors that confirm the discrimination capability among the three tissue types, based on their autofluorescence lifetime. In Figure 3C,D the three tissue types are depicted in dark red, green‐yellow, or blue/light blue, demonstrating a lifetime decrease in both emission channels when moving from the left (ICC region) to the right side of the image (healthy region). The results obtained from data acquired in the two channels are quite similar, as confirmed by the autofluorescence lifetime profiles reported in Figure 3B, where the behaviors of the autofluorescence lifetime along the dashed line in Figure 3A for CH1 (plain line) and CH2 (dashed line) are almost coincident. The *τ*_phase_ for both channels increased significantly in ICC metastasis when compared to both surgical margin and healthy liver. On the other hand, the values measured in surgical margin are similar to those ones found in healthy liver, as reported in the histograms in Figure 3G for CH1 and 3 h for CH2. More in detail, the average *τ*_phase_ of the ICC region was found equal to 4.2 ± 0.2 ns and 4.6 ± 0.3 ns in CH1 and CH2, respectively. The average values of *τ*_phase_ for the surgical margin and the healthy liver were found equal to 2.1 ± 0.2 ns and 2.2 ± 0.1 ns in CH1, and to 2.0 ± 0.2 ns and 2.3 ± 0.3 ns in CH2, respectively. These values (Table 1 for a schematic view) demonstrate a general homogeneity in the healthy liver environment, independently from the proximity of the tumor.

### 3.2 Gastrointestinal stromal tumor and pancreatic tumor

**FIGURE 4:**
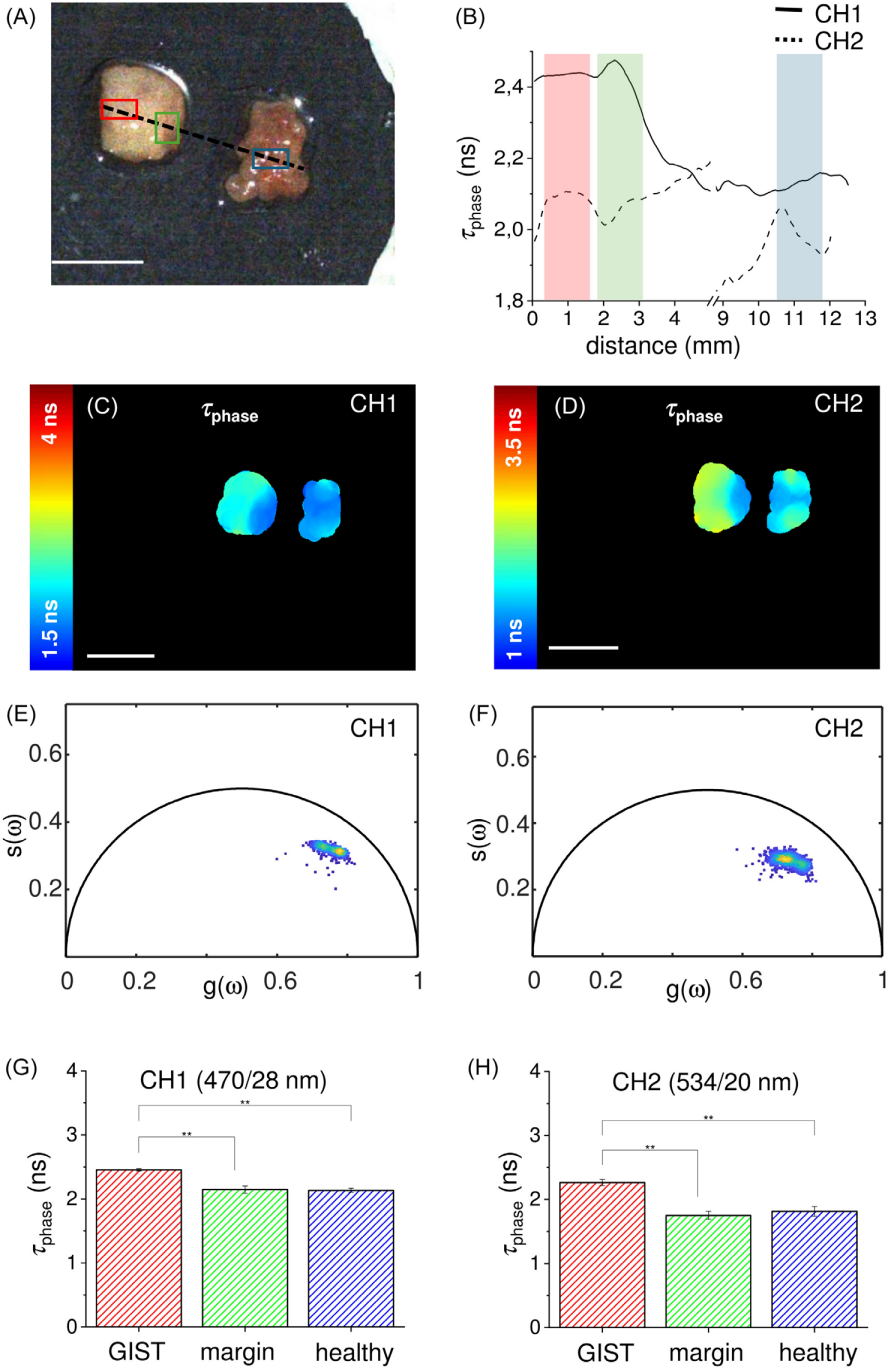
(a) White light image of the GIST sample with superimposed drawings of healthy tissue ROI (cyan), surgical margin ROI (green), tumor ROI (red), and ROIs crossing profile (black dashed line). (b) Profile of the autofluorescence lifetime values of *τ*_phase_ measured along the crossing profile in (a) for the channel 470/28 nm (CH1, black line) and 534/20 nm (CH2, black dashed line). Autofluorescence lifetime maps for the channel 470/28 nm (CH1, c) and 534/20 nm (CH2, d), with the corresponding phasor plots for the channel 470/28 nm (CH1, e) and 534/20 nm (CH2, f). Scale bars = 10 mm. (g, h) Histograms of the autofluorescence lifetime values of *τ*_phase_ acquired in the two spectral channels and averaged over ROI of tumor region (dark grey), surgical margin (light grey) and healthy tissue distant from the lesion (grey dashed lines). Values are reported as mean ± standard deviation, and the student *t*‐test show the statistically significant differences between those ROIs (*p*‐values <.001).

**FIGURE 5:**
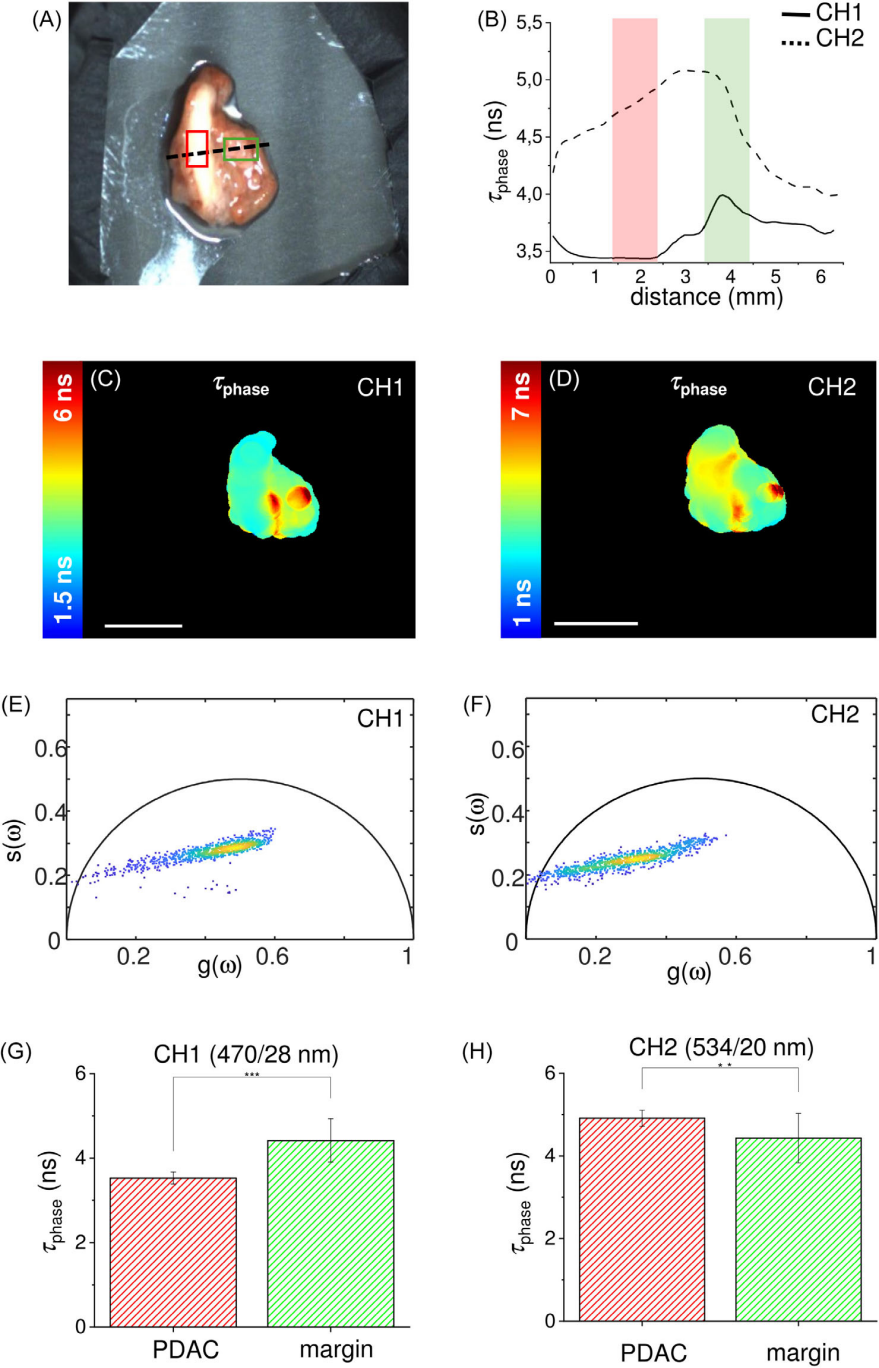
(a) White light image of the PDAC sample with superimposed drawings of surgical margin ROI (green), tumor ROI (red), and ROIs crossing profile (black dashed line). (b) Profile of the autofluorescence lifetime values of *τ*_phase_ measured along the crossing profile in (a) for the channel 470/28 nm (CH1, black line) and 534/20 nm (CH2, black dashed line). Autofluorescence lifetime maps for the channel 470/28 nm (CH1, c) and 534/20 nm (CH2, e), with the corresponding phasor plots for the channel 470/28 nm (CH1, f) and 534/20 nm (CH2, g). Scale bars = 10 mm. (g, h) Histograms of the autofluorescence lifetime values of *τ*_phase_ acquired in the two spectral channels and averaged over ROI of tumor region (dark grey) and surgical margin (light grey). Values are reported as mean ± standard deviation, and the student *t*‐test show the statistically significant differences between those ROIs (*p*‐values <.001).

Figures 4 and 5 present the results obtained in other two examples, corresponding to two different clinical cases and organs. In particular, Figure 4 shows the results obtained on a tissue sample of GIST, a tumor that has the morphological characteristic to evert from the proliferation site [34]. The white light image (Figure 4A) shows two distinct biopsies: the GIST with its surgical margin on the left and the corresponding healthy mucosa on the right. In this case the margin is not distinguishable by naked eye, while it is easily detected based on the measured autofluorescence lifetime values. As shown in the maps in Figure 4C,D, the variation of the autofluorescence lifetime across the samples (about 0.5 ns on both channels), despite being small, it is sufficiently high to provide a clear contrast between different tissue types. In particular, the surgical margin region can be distinguished from the tumor region, as demonstrated by the colors shown in the autofluorescence lifetime maps of Figure 4C,D, where the right side of the tissue fragment on the left (surgical margin) shows a color that is different from the left side of the same fragment (GIST) and similar to the tissue fragment on the right (healthy tissue) on both detection channels. This result is confirmed by the corresponding phasor plots (Figure 3E,F for CH1 and CH2, respectively), where a similar behavior for the two detection channels is shown. In both graphs the phasor points are distributed around two areas that are quite close each other but still consistent with a bimodal distribution, that confirm the discrimination capability between the GIST and its surgical margin based on their autofluorescence lifetime, as well as the similarity of the surgical margin with healthy tissue, making the corresponding distributions indistinguishable in the phasor domain. This result agrees with the autofluorescence lifetime profile reported in Figure 4B, where the behaviors of the autofluorescence lifetime along the dashed line in Figure 4A for CH1 (plain line) and CH2 (dashed line) exhibit a monotonic decrease when passing from the GIST region to the surgical margin region, face to an almost constant behavior when passing from surgical margin region to the healthy tissue region. The ROIs analysis provided further confirmation of this result, as reported in the histograms in Figure 4G for CH1 and 4 h for CH2. In particular, the *τ*_phase_ for GIST region was found equal to 2.4 ± 0.01 ns and 2.3 ± 0.04 ns in CH1 and CH2, respectively. The surgical margin exhibits shorter autofluorescence lifetime with respect to the tumor, with a *τ*_phase_ of 2.1 ± 0.5 ns in CH1, and 1.7 ± 0.06 ns in CH2. The healthy tissue exhibits *τ*_phase_ values comparable to those measured in the surgical margin ROI (see Table 1 for details).

The last clinical case here reported (Figure 5) is a PDAC. In this specific class of cancers, it is typically difficult performing a visual margin assessment [5, 12]. Nevertheless, in our sample the tumor was visually distinguishable (Figure 5A) because is light brown colored, while the surgical margin presents a light red color. In this example, we did not examine a corresponding healthy sample, limiting the analysis to PDAC and surgical margin regions. The autofluorescence lifetime map for CH1, represented in Figure 5C, shows a slightly shorter autofluorescence lifetime in PDAC region (on the left) in comparison to the surgical margin (on the right). On the other hand, this difference is reversed on CH2, where the PDAC region exhibits a slightly longer autofluorescence lifetime than the surgical margin region (Figure 5D). This result is confirmed by the corresponding phasor plots (Figure 5E,F for CH1 and CH2, respectively), where a different behavior for the two detection channels is shown. In particular, while the two phasor clouds are distributed along the same line, the barycentre of the two distributions are located in different regions for the two channels, confirming the channel‐dependent result shown in the autofluorescence lifetime maps. The discrimination of two distinct regions in the phasor domain is more difficult here than in the previous examples, in agreement with the small difference observed in terms of autofluorescence lifetime between the two tissue types. Nevertheless, a bimodal distribution can be determined by looking at the two phasor clouds, especially that one in CH2. The margin delineation capability of the method is further confirmed by the autofluorescence lifetime profiles reported in Figure 5B, where the behaviors of the autofluorescence lifetime along the dashed line in Figure 5A for CH1 (plain line) and CH2 (dashed line) are reported. Here, an opposite trend between the two channels is found when comparing surgical margin and PDAC tissue, with surgical margin region showing a longer lifetime in CH1 and a shorter lifetime in CH2 when compared to the corresponding values measured in PDAC tissue. This result is consistent with the results obtained from the ROIs analysis, where we observed a statistically significant difference in terms of autofluorescence lifetime between the examined tissue types, consisting of a respective decrease/increase of the autofluorescence lifetime of PDAC cells on CH1/CH2, when compared to the surgical margin (Figure 5G,H). The *τ*_phase_ of PDAC calculated from data acquired in CH1 was 3.5 ± 0.1 ns, while in CH2 was 4.9 ± 0.2 ns. On the other hand, the surgical margin region shows a *τ*_phase_ of 4.4 ± 0.5 ns in CH1 and 4.4 ± 0.6 ns in CH2. In other words, the lifetime shift between surgical margin and tumor region exhibits opposite signs in the two channels, with a shorter autofluorescence lifetime of the PDAC with respect to its margin in CH1, face to a longer lifetime in CH2.

## 4 DISCUSSION

The suitability of our method to produce label free autofluorescence lifetime maps in real time was previously demonstrated for both biological tissue [26] and cultural heritage specimens [28]. Here, we tested the use of autofluorescence lifetime imaging for discriminating tumor lesions against surgical margin in four cases of freshly excised tumor tissue biopsies. The results show the capabilities of autofluorescence lifetime measurements in delineating cancer margins in every examined specimen. More in detail, in the HCC specimen (Figure 2) the surgical margin exhibits a longer autofluorescence lifetime compared to the tumor region. The phasor analysis yielded a *τ*_phase_ difference of 0.6 ns between the two regions for both the 470 nm band (CH1) and the 534 nm band (CH2). Although a single instance is insufficient to establish a definitive trend, these findings corroborate existing literature, which suggests that HCC typically exhibits a shorter mean fluorescence lifetime compared to surrounding perilesional tissue in rats [35]. Even if the *τ*_phase_ difference between surgical margin and HCC is consistent on the two detection channels, both regions exhibit significantly higher values of *τ*_phase_ in CH2 than in CH1. On the other hand, a similar trend was not observed in healthy tissue, as the corresponding *τ*_phase_ values are slightly lower on CH2 with respect to CH1. Considering the excitation at 375 nm, the major endogenous fluorophore contributing to the signal in the 470 nm band (CH1) should be NAD(P)H, whereas both NAD(P)H and other fluorophores (i.e., flavins) co‐contribute to the signal detected in the 534 nm band (CH2). The different trend between healthy tissue and the other two regions observed in CH2 could be probably due to the different balance between NAD(P)H and other fluorophores with an emission peak in the 534 nm band occurring in both surgical margin and HCC region. In the ICC (Figure 3), we found a notable increase of autofluorescence lifetime (approximately 2 ns) compared to the healthy tissue, as both the surgical margin and the healthy liver exhibit comparable autofluorescence lifetimes on both detection channels. Like HCC, which typically occurs in pre‐cancerous liver inflammation or hepatitis [36], ICC usually develops in a background of chronic inflammation, which causes cholestasis and resultant cholangiocyte injury [37], making the divergence in healthy tissue behavior plausible due to the internal variability inherent in the inflammatory state. Similarly, in the GIST specimen (Figure 4), the healthy mucosa displays *τ*_phase_ values comparable to those of the surgical margin on both channels, demonstrating that the delineated surgical margin exhibits metabolic features more aligned to those of healthy tissue than to tumoral ones. On the other hand, differently from ICC and GIST, PDAC (Figure 5) showed a distinct trend related to the detection channel: the autofluorescence lifetime shift between surgical margin and tumor region exhibits opposite signs in the two channels, with a shorter autofluorescence lifetime of the PDAC with respect to its margin in CH1, face to a longer lifetime in CH2. Similar to HCC, this result could be probably due to the contribution of fluorophores with an emission peak in CH2 that dominates over NAD(P)H in this spectral region. On the other hand, the homogeneity of the results obtained for ICC and GIST in the two spectral channels, agree with the hypothesis of an individual main endogenous fluorophore (NAD(P)H) as major contributor to the detected signal in both CH1 and CH2 [38]. Further additional measurements, employing a system with two (or three) different excitation wavelengths, for targeting specific fluorophores, could help in clarifying this point.

The presented approach offers a large potential for future clinical translation, as the acquisition is performed under bright background conditions, thanks to an asynchronous autofluorescence acquisition with an externally modulated white light source. The integration in an optical fiber probe offers greater flexibility than traditional imaging modalities, in terms of both ease of sampling and integration within existing clinical systems. Further, the phasors approach for data analysis, based on FFT of the decay function, allows real‐time processing and identification of pixel‐based lifetime characteristics without prior knowledge of the exact contribution of different components [29, 30], facilitating the recognition of distinct lifetime subsets with subcellular precision [39]. The clinical utility of the technology described here is currently constrained to superficial information and macroscopic measurements. This is primarily due to the limited penetration depth of the 375 nm excitation light, limited to few hundreds of μm. Moreover, when dealing with ex vivo specimens, there's a notable absence of tissue oxygenation, resulting in a blend of various oxygen‐responsive biochemical species. This presents both a challenge and an opportunity for refining our technique that still needs to be tested in vivo, that is, on the animal model, to better figure out the metabolic information that can be extracted with this method. Despite the large amount of work to be done before translating this technology to the clinics, our vision is to deploy this technology intraoperatively, particularly in resection surgeries like robot‐assisted colorectal procedures.” In fact, the obtained results have shown that the proposed method provided sufficiently high accuracy to discriminate tumor and delineate its surgical margin in all the examined cases, together with the advantage of real‐time processing, hence with a significantly high potential for clinical translation. In summary, the combination of the easy handling provided by optical fiber, the diagnostic accuracy provided by autofluorescence lifetime detection, and the real‐time processing offered by phasors analysis allows rapid acquisition and quantitative processing of autofluorescence lifetime data in ex vivo human tumor biopsies, paving the way for its validation in a clinical scenario.

## 5 CONCLUSION

In conclusion, this pilot study presented the test and exploitation of an optical setup for autofluorescence lifetime measurements with a great potential for clinical translation. We realized single‐point fiber‐based fluorescence lifetime imaging in real‐time and under bright illumination of the FOV for the rapid characterization and delineation of healthy surgical margins with respect to the cancer mass in four different clinically relevant use cases. The obtained results demonstrated that our approach based on real‐time autofluorescence lifetime measurements can successfully delineate cancer margins and better characterize the lesion in every examined specimen. This approach can be further developed and potentially tailored to different clinical applications, as the system is based on a flexible optical fiber bundle and hence it is potentially compatible with various standard clinical imaging modalities. Future work will aim to multiplex the excitation wavelengths used, to validate the method on a dataset with a significantly higher statistical relevance by increasing the number of examined samples, as well to design a more a compact and portable instrument for facilitating the integration with existing clinical instruments.

## AUTHOR CONTRIBUTIONS

R.C., J.L., L.A. and A.T. were involved in conceptualization, investigation, writing (original draft, review and editing). D.S., L.T. and E.B. were involved in sample preparation, data acquisition, data analysis and writing (review and editing). S.B., C.A. and A.T. were involved in specimen sampling and surgical procedures. S.P. and A.T. were involved in ethics. R.C. and A.T. were involved in project management.

## CONFLICT OF INTEREST STATEMENT

The authors declare no financial or commercial conflict of interest.

## Data Availability

The data that support the findings of this study are available from the corresponding author upon reasonable request.
